# Bis[1,3-bis­(benzimidazol-2-yl)-2-oxapropane]copper(II)–picrate–dimethyl­formamide (1/2/4)

**DOI:** 10.1107/S1600536808036829

**Published:** 2008-11-13

**Authors:** Ruirui Yun, Wei Ying, Baoliang Qi, Xuyang Fan, Huilu Wu

**Affiliations:** a School of Chemical and Biological Engineering, Lanzhou Jiaotong University, Lanzhou 730070, People’s Republic of China; b College of Chemistry and Chemical Engineering, Lanzhou University, Lanzhou 730000, People’s Republic of China

## Abstract

In the title compound, [Cu(C_16_H_14_N_4_O)_2_](C_6_H_2_N_3_O_7_)_2_·4C_3_H_7_NO, the Cu^II^ ion is located on a crystallographic inversion center and is coordinated in a distorted octa­hedral environment by four N atoms and two O atoms forming two long Cu—O bonds. One of the unique dimethyl­formamide solvent mol­ecules is disordered over two sites with occupancies of 0.715 (6) and 0.285 (6). The crystal structure is stabilized by inter­molecular N—H⋯O hydrogen bonds.

## Related literature

For the analagous Zn(II) diperchlorate complex, see: Zhou & Yang (2006 ▶).
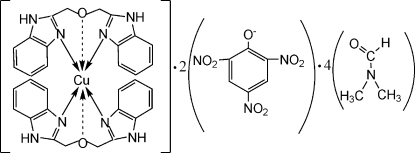


## Experimental

### 

#### Crystal data

[Cu(C_16_H_14_N_4_O)_2_](C_6_H_2_N_3_O_7_)_2_·4C_3_H_7_NO
*M* *_r_* = 1368.77
Triclinic, 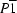

*a* = 10.9656 (7) Å
*b* = 12.6028 (12) Å
*c* = 13.4100 (9) Å
α = 65.746 (2)°
β = 88.629 (2)°
γ = 65.187 (2)°
*V* = 1508.8 (2) Å^3^
*Z* = 1
Mo *K*α radiation
μ = 0.46 mm^−1^
*T* = 293 (2) K
0.28 × 0.21 × 0.11 mm

#### Data collection

Rigaku R-AXIS Spider diffractometer
Absorption correction: multi-scan (*ABSCOR*; Higashi, 1995 ▶) *T*_min_ = 0.883, *T*_max_ = 0.952
12429 measured reflections
5605 independent reflections
3363 reflections with *I* > 2σ(*I*)
*R*_int_ = 0.078

#### Refinement

*R*[*F* ^2^ > 2σ(*F* ^2^)] = 0.075
*wR*(*F* ^2^) = 0.232
*S* = 1.01
5605 reflections
457 parameters
18 restraints
H atoms treated by a mixture of independent and constrained refinement
Δρ_max_ = 0.78 e Å^−3^
Δρ_min_ = −1.09 e Å^−3^

### 

Data collection: *RAPID-AUTO* (Rigaku/MSC, 2004 ▶); cell refinement: *RAPID-AUTO*; data reduction: *RAPID-AUTO*; program(s) used to solve structure: *SHELXS97* (Sheldrick, 2008 ▶); program(s) used to refine structure: *SHELXL97* (Sheldrick, 2008 ▶); molecular graphics: *PLATON* (Spek, 2003 ▶); software used to prepare material for publication: *SHELXTL* (Sheldrick, 2008 ▶).

## Supplementary Material

Crystal structure: contains datablocks global, I. DOI: 10.1107/S1600536808036829/lh2715sup1.cif

Structure factors: contains datablocks I. DOI: 10.1107/S1600536808036829/lh2715Isup2.hkl

Additional supplementary materials:  crystallographic information; 3D view; checkCIF report

## Figures and Tables

**Table:** 

Cu—N3	1.979 (3)
Cu—N1	1.992 (4)
Cu—O1	2.583 (3)

**Table:** 

N3—Cu—N3^i^	180
N3—Cu—N1	87.55 (15)
N3—Cu—N1^i^	92.45 (15)
N1—Cu—N1^i^	180
N3—Cu—O1^i^	106.54 (12)
N1—Cu—O1^i^	106.14 (13)
N3—Cu—O1	73.46 (12)
N1—Cu—O1	73.86 (13)
O1^i^—Cu—O1	180

Symmetry code: (i) 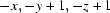.

**Table 2:** Hydrogen-bond geometry (Å, °)

*D*—H⋯*A*	*D*—H	H⋯*A*	*D*⋯*A*	*D*—H⋯*A*
N2—H2*N*⋯O6^ii^	0.87 (5)	1.95 (4)	2.709 (5)	145 (6)
N2—H2*N*⋯O5^ii^	0.87 (5)	2.41 (5)	3.104 (6)	138 (6)
N4—H4*N*⋯O9^iii^	0.87 (5)	1.92 (2)	2.753 (6)	162 (5)

Symmetry codes: (ii) 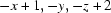; (iii) 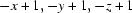.

**Fig. 1.**
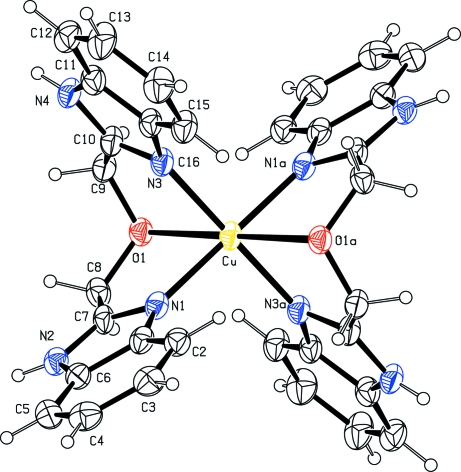
The cation of the title compound showing displacement ellipsoids at the 30% probability level [symmetry code: (a) -x, -y+1, -x+1].

## supplementary crystallographic information

### Comment

The asymmetric unit of the title compound consists of half of a di[1,3-bis(benzimidazol-2-yl)-2-oxopropane] copper(II) cation (Fig.1), one picrate anion and two molecules of DMF. The Cu^II^ ion is six-coordinated with a N_4_O_2_ ligand set. The obb (1,3-bis(benzimidazol-2-yl)-2-oxopropane) ligand acts as a tridentate donor. The coordination geometry of the Cu^II^ may be best described as distorted octahedral. This geometry is assumed by the Cu^II^ to relieve the steric crowding. The equatorial plane is occupied by four N atoms of two benzimidazolyl groups. The axial positions are occuppied two O atoms.  The crystal structure is stabilized by intermolecular N-H···O hydrogen bonds.

### Experimental

To a stirred solution of 1,3-bis(benzimidazol-2-yl)-2-oxopropane (0.139 g, 0.5 mmol) in hot MeOH (15 ml) was added Cu(C_6_H_2_N_3_O_7_)_2_ (0.130 g, 0.25 mmol) in MeOH (5 ml). A green crystalline product formed rapidly. The precipitate was filtered off, washed with MeOH and absolute Et_2_O, and dried *in vacuo*. The dried precipitate was dissolved in DMF resulting in a green solution. The green crystals suitable for X-ray diffraction studies were obtained by ether diffusion into DMF after three days at room temperature. Yield, 0.106 g (66%). (found: C, 49.23; H, 4.37; N,18.58. Calcd. for C56H60N18O20Cu: C, 49.14; H, 4.42; N, 18.42)

### Refinement

All H atoms were found in difference electron maps and were subsequently refined in a riding-model approximation with C—H distances ranging from 0.93 to 0.96 Å and *U*_iso_(H) = 1.2 *U*_eq_(C). The H atoms bonded to N atoms were refined independently with the distance constraint of N-H = 0.86 (1)Å. One of the unique DMF solvent molecules is disorderd over two sites with refined occupancies 0.715 (6) and 0.285 (6). The minor component was refined isotropically and constrained to be geometrically similar to the major component using the SAME instruction in SHELXL (Sheldrick, 2008).

### Crystal data

**Table:**

[Cu(C_16_H_14_N_4_O)_2_](C_6_H_2_N_3_O_7_)_2_·4C_3_H_7_NO	*Z* = 1
*M**_r_* = 1368.77	*F*_000_ = 711
Triclinic, *P*1	*D*_x_ = 1.506 Mg m^−^^3^
Hall symbol: -P 1	Mo *K*α radiation λ = 0.71073 Å
*a* = 10.9656 (7) Å	Cell parameters from 5605 reflections
*b* = 12.6028 (12) Å	θ = 3.0–25.5º
*c* = 13.4100 (9) Å	µ = 0.46 mm^−^^1^
α = 65.746 (2)º	*T* = 293 (2) K
β = 88.629 (2)º	Block, green
γ = 65.187 (2)º	0.28 × 0.21 × 0.11 mm
*V* = 1508.8 (2) Å^3^	

### Data collection

**Table:**

Rigaku R-AXIS Spider diffractometer	5605 independent reflections
Radiation source: fine-focus sealed tube	3363 reflections with *I* > 2σ(*I*)
Monochromator: graphite	*R*_int_ = 0.078
*T* = 293(2) K	θ_max_ = 25.5º
φ and ω scans	θ_min_ = 3.0º
Absorption correction: multi-scan(ABSCOR; Higashi, 1995)	*h* = −13→13
*T*_min_ = 0.883, *T*_max_ = 0.952	*k* = −15→15
12429 measured reflections	*l* = −16→15

### Refinement

**Table:**

Refinement on *F*^2^	Secondary atom site location: difference Fourier map
Least-squares matrix: full	Hydrogen site location: inferred from neighbouring sites
*R*[*F*^2^ > 2σ(*F*^2^)] = 0.075	H atoms treated by a mixture of independent and constrained refinement
*wR*(*F*^2^) = 0.232	*w* = 1/[σ^2^(*F*_o_^2^) + (0.1327*P*)^2^] where *P* = (*F*_o_^2^ + 2*F*_c_^2^)/3
*S* = 1.02	(Δ/σ)_max_ < 0.001
5605 reflections	Δρ_max_ = 0.78 e Å^−^^3^
457 parameters	Δρ_min_ = −1.09 e Å^−^^3^
18 restraints	Extinction correction: SHELXL97 (Sheldrick, 2008), Fc^*^=kFc[1+0.001xFc^2^λ^3^/sin(2θ)]^-1/4^
Primary atom site location: structure-invariant direct methods	Extinction coefficient: 0.009 (3)

### Special details

**Table:**

Geometry. All e.s.d.'s (except the e.s.d. in the dihedral angle between two l.s. planes) are estimated using the full covariance matrix. The cell e.s.d.'s are taken into account individually in the estimation of e.s.d.'s in distances, angles and torsion angles; correlations between e.s.d.'s in cell parameters are only used when they are defined by crystal symmetry. An approximate (isotropic) treatment of cell e.s.d.'s is used for estimating e.s.d.'s involving l.s. planes.
Refinement. Refinement of *F*^2^ against ALL reflections. The weighted *R*-factor *wR* and goodness of fit *S* are based on *F*^2^, conventional *R*-factors *R* are based on *F*, with *F* set to zero for negative *F*^2^. The threshold expression of *F*^2^ > σ(*F*^2^) is used only for calculating *R*-factors(gt) *etc*. and is not relevant to the choice of reflections for refinement. *R*-factors based on *F*^2^ are statistically about twice as large as those based on *F*, and *R*- factors based on ALL data will be even larger.

### Fractional atomic coordinates and isotropic or equivalent isotropic displacement parameters (Å^2^)

**Table:**

	*x*	*y*	*z*	*U*_iso_*/*U*_eq_	Occ. (<1)
Cu	0.0000	0.5000	0.5000	0.0414 (3)	
O1	0.1409 (3)	0.5704 (3)	0.5882 (2)	0.0445 (8)	
O2	−0.1106 (4)	0.1849 (4)	0.7815 (3)	0.0747 (12)	
O3	−0.0335 (4)	−0.0186 (5)	0.8206 (3)	0.0701 (11)	
O4	0.4289 (4)	−0.3097 (4)	1.0041 (3)	0.0651 (11)	
O5	0.4919 (4)	−0.2964 (4)	1.1479 (3)	0.0816 (14)	
O6	0.4312 (3)	−0.0496 (4)	1.1048 (3)	0.0577 (10)	
O7	0.3068 (6)	0.1965 (6)	1.0630 (6)	0.151 (3)	
O8	0.1451 (5)	0.3258 (4)	0.9307 (4)	0.0838 (13)	
N1	0.1462 (3)	0.3364 (4)	0.6167 (3)	0.0418 (9)	
N2	0.3339 (4)	0.2162 (4)	0.7450 (3)	0.0440 (10)	
N3	0.1426 (3)	0.5132 (4)	0.4093 (3)	0.0434 (10)	
N4	0.3198 (4)	0.5527 (5)	0.3577 (3)	0.0493 (10)	
N5	−0.0233 (4)	0.0710 (6)	0.8286 (3)	0.0577 (12)	
N6	0.4176 (4)	−0.2487 (5)	1.0584 (3)	0.0574 (12)	
N7	0.2262 (4)	0.2166 (5)	0.9921 (3)	0.0593 (12)	
C1	0.1934 (4)	0.2071 (5)	0.6375 (4)	0.0431 (11)	
C2	0.1446 (5)	0.1495 (5)	0.5898 (4)	0.0488 (12)	
H2	0.0674	0.1994	0.5343	0.059*	
C3	0.2141 (5)	0.0169 (6)	0.6273 (4)	0.0561 (13)	
H3	0.1823	−0.0239	0.5980	0.067*	
C4	0.3320 (5)	−0.0576 (6)	0.7088 (4)	0.0586 (14)	
H4	0.3773	−0.1471	0.7319	0.070*	
C5	0.3832 (5)	−0.0029 (5)	0.7558 (4)	0.0490 (12)	
H5	0.4619	−0.0528	0.8098	0.059*	
C6	0.3118 (4)	0.1294 (5)	0.7189 (3)	0.0436 (11)	
C7	0.2347 (4)	0.3364 (5)	0.6822 (4)	0.0433 (12)	
C8	0.2230 (4)	0.4562 (5)	0.6871 (4)	0.0468 (12)	
H8A	0.1828	0.4627	0.7508	0.056*	
H8B	0.3134	0.4503	0.6965	0.056*	
C9	0.2173 (4)	0.6123 (5)	0.5063 (4)	0.0481 (12)	
H9A	0.3083	0.5841	0.5424	0.058*	
H9B	0.1733	0.7067	0.4674	0.058*	
C10	0.2273 (4)	0.5579 (5)	0.4249 (4)	0.0464 (12)	
C11	0.2956 (4)	0.5003 (5)	0.2916 (4)	0.0498 (13)	
C12	0.3633 (5)	0.4699 (6)	0.2106 (4)	0.0596 (15)	
H12	0.4351	0.4895	0.1874	0.072*	
C13	0.3170 (5)	0.4088 (7)	0.1670 (5)	0.0686 (17)	
H13	0.3598	0.3854	0.1134	0.082*	
C14	0.2084 (5)	0.3810 (6)	0.2004 (4)	0.0662 (16)	
H14	0.1808	0.3396	0.1687	0.079*	
C15	0.1411 (5)	0.4134 (6)	0.2791 (4)	0.0564 (14)	
H15	0.0686	0.3947	0.3014	0.068*	
C16	0.1863 (4)	0.4754 (5)	0.3240 (4)	0.0444 (11)	
C17	0.0969 (4)	0.0407 (5)	0.8985 (4)	0.0480 (12)	
C18	0.2005 (5)	−0.0854 (5)	0.9460 (4)	0.0496 (13)	
H18	0.1943	−0.1493	0.9315	0.060*	
C19	0.3118 (4)	−0.1151 (5)	1.0143 (4)	0.0496 (13)	
C20	0.3311 (4)	−0.0229 (5)	1.0410 (3)	0.0456 (12)	
C21	0.2194 (5)	0.1088 (5)	0.9812 (4)	0.0472 (12)	
C22	0.1076 (4)	0.1381 (5)	0.9134 (4)	0.0488 (13)	
H22	0.0392	0.2234	0.8776	0.059*	
O9	0.5277 (3)	0.3378 (4)	0.6011 (3)	0.0581 (10)	
N8	0.5913 (4)	0.1433 (4)	0.6002 (3)	0.0551 (12)	
C23	0.6860 (5)	0.0045 (5)	0.6489 (4)	0.0723 (18)	
H23A	0.7387	−0.0147	0.5950	0.087*	
H23B	0.6355	−0.0454	0.6710	0.087*	
H23C	0.7461	−0.0176	0.7128	0.087*	
C24	0.4876 (5)	0.1923 (6)	0.5051 (4)	0.0651 (16)	
H24A	0.4192	0.2779	0.4908	0.078*	
H24B	0.4459	0.1350	0.5211	0.078*	
H24C	0.5295	0.1961	0.4408	0.078*	
C25	0.6028 (5)	0.2181 (5)	0.6400 (4)	0.0551 (14)	
H25	0.6710	0.1799	0.7010	0.066*	
O10	0.1119 (11)	0.8119 (10)	0.6125 (7)	0.161 (4)	0.715 (6)
N9	0.1418 (5)	0.6993 (7)	0.7922 (6)	0.068 (2)	0.715 (6)
C26	0.2883 (6)	0.6379 (9)	0.7915 (9)	0.095 (4)	0.715 (6)
H26A	0.3044	0.6737	0.7170	0.143*	0.715 (6)
H26B	0.3205	0.5457	0.8187	0.143*	0.715 (6)
H26C	0.3360	0.6541	0.8384	0.143*	0.715 (6)
C27	0.0966 (8)	0.6585 (9)	0.8996 (6)	0.081 (3)	0.715 (6)
H27A	−0.0009	0.7062	0.8888	0.122*	0.715 (6)
H27B	0.1386	0.6758	0.9493	0.122*	0.715 (6)
H27C	0.1225	0.5670	0.9309	0.122*	0.715 (6)
C28	0.0578 (8)	0.7873 (8)	0.6984 (6)	0.071 (3)	0.715 (6)
H28A	−0.0359	0.8292	0.6950	0.085*	0.715 (6)
O10A	0.3069 (17)	0.6919 (19)	0.6726 (13)	0.100*	0.285 (6)
N9A	0.1822 (19)	0.667 (3)	0.8055 (15)	0.100*	0.285 (6)
C26A	0.303 (2)	0.552 (2)	0.8825 (17)	0.100*	0.285 (6)
H26D	0.3777	0.5331	0.8430	0.150*	0.285 (6)
H26E	0.2838	0.4784	0.9143	0.150*	0.285 (6)
H26F	0.3281	0.5688	0.9406	0.150*	0.285 (6)
C27A	0.0588 (19)	0.700 (3)	0.8533 (19)	0.100*	0.285 (6)
H27D	−0.0151	0.7755	0.7973	0.150*	0.285 (6)
H27E	0.0739	0.7182	0.9135	0.150*	0.285 (6)
H27F	0.0364	0.6282	0.8806	0.150*	0.285 (6)
C28A	0.1935 (19)	0.729 (2)	0.7029 (14)	0.100*	0.285 (6)
H28B	0.1175	0.8016	0.6525	0.120*	0.285 (6)
H2N	0.400 (4)	0.199 (6)	0.792 (4)	0.08 (2)*	
H4N	0.374 (5)	0.587 (5)	0.357 (4)	0.069 (18)*	

### Atomic displacement parameters (Å^2^)

**Table:**

	*U*^11^	*U*^22^	*U*^33^	*U*^12^	*U*^13^	*U*^23^
Cu	0.0295 (4)	0.0509 (6)	0.0466 (5)	−0.0148 (4)	0.0089 (3)	−0.0273 (4)
O1	0.0332 (15)	0.051 (2)	0.0461 (17)	−0.0136 (15)	0.0083 (14)	−0.0237 (16)
O2	0.045 (2)	0.073 (3)	0.077 (3)	−0.011 (2)	−0.0100 (19)	−0.021 (2)
O3	0.058 (2)	0.081 (3)	0.070 (2)	−0.029 (2)	−0.0037 (19)	−0.033 (2)
O4	0.063 (2)	0.059 (3)	0.068 (2)	−0.0146 (19)	0.0012 (18)	−0.037 (2)
O5	0.081 (3)	0.064 (3)	0.064 (2)	−0.002 (2)	−0.032 (2)	−0.026 (2)
O6	0.0501 (19)	0.060 (2)	0.0532 (19)	−0.0160 (17)	−0.0023 (16)	−0.0245 (18)
O7	0.136 (5)	0.082 (4)	0.197 (6)	0.010 (3)	−0.097 (5)	−0.082 (4)
O8	0.084 (3)	0.059 (3)	0.088 (3)	−0.019 (2)	−0.011 (2)	−0.026 (2)
N1	0.0338 (18)	0.051 (3)	0.047 (2)	−0.0186 (18)	0.0123 (17)	−0.027 (2)
N2	0.0352 (19)	0.054 (3)	0.044 (2)	−0.0163 (19)	0.0124 (18)	−0.027 (2)
N3	0.0315 (17)	0.054 (3)	0.045 (2)	−0.0156 (18)	0.0086 (16)	−0.027 (2)
N4	0.0325 (19)	0.058 (3)	0.059 (2)	−0.019 (2)	0.0144 (19)	−0.028 (2)
N5	0.041 (2)	0.080 (4)	0.047 (2)	−0.023 (2)	0.0048 (19)	−0.027 (3)
N6	0.052 (2)	0.059 (3)	0.057 (2)	−0.015 (2)	0.007 (2)	−0.032 (2)
N7	0.051 (2)	0.063 (3)	0.048 (2)	−0.007 (2)	−0.002 (2)	−0.030 (2)
C1	0.037 (2)	0.055 (3)	0.045 (2)	−0.021 (2)	0.015 (2)	−0.029 (2)
C2	0.043 (2)	0.058 (3)	0.048 (3)	−0.022 (2)	0.012 (2)	−0.026 (3)
C3	0.061 (3)	0.063 (4)	0.053 (3)	−0.030 (3)	0.018 (3)	−0.033 (3)
C4	0.067 (3)	0.054 (4)	0.059 (3)	−0.026 (3)	0.025 (3)	−0.030 (3)
C5	0.045 (2)	0.058 (3)	0.042 (2)	−0.015 (2)	0.009 (2)	−0.028 (2)
C6	0.037 (2)	0.048 (3)	0.041 (2)	−0.013 (2)	0.013 (2)	−0.022 (2)
C7	0.0260 (19)	0.061 (3)	0.042 (2)	−0.012 (2)	0.0078 (18)	−0.030 (2)
C8	0.039 (2)	0.058 (3)	0.044 (2)	−0.017 (2)	0.002 (2)	−0.027 (2)
C9	0.035 (2)	0.059 (3)	0.053 (3)	−0.021 (2)	0.010 (2)	−0.028 (3)
C10	0.031 (2)	0.058 (3)	0.053 (3)	−0.017 (2)	0.009 (2)	−0.030 (2)
C11	0.036 (2)	0.057 (3)	0.049 (3)	−0.011 (2)	0.009 (2)	−0.027 (3)
C12	0.043 (3)	0.073 (4)	0.062 (3)	−0.021 (3)	0.021 (2)	−0.036 (3)
C13	0.048 (3)	0.096 (5)	0.075 (3)	−0.024 (3)	0.027 (3)	−0.059 (4)
C14	0.057 (3)	0.085 (5)	0.070 (3)	−0.026 (3)	0.021 (3)	−0.052 (3)
C15	0.041 (2)	0.070 (4)	0.062 (3)	−0.021 (3)	0.014 (2)	−0.038 (3)
C16	0.031 (2)	0.053 (3)	0.047 (2)	−0.012 (2)	0.0093 (19)	−0.027 (2)
C17	0.038 (2)	0.059 (3)	0.043 (2)	−0.018 (2)	0.008 (2)	−0.023 (2)
C18	0.046 (3)	0.064 (4)	0.041 (2)	−0.027 (3)	0.014 (2)	−0.023 (2)
C19	0.040 (2)	0.057 (3)	0.043 (2)	−0.010 (2)	0.005 (2)	−0.026 (2)
C20	0.041 (2)	0.052 (3)	0.033 (2)	−0.013 (2)	0.008 (2)	−0.017 (2)
C21	0.047 (3)	0.052 (3)	0.043 (2)	−0.017 (2)	0.016 (2)	−0.027 (2)
C22	0.036 (2)	0.058 (3)	0.041 (2)	−0.010 (2)	0.007 (2)	−0.022 (2)
O9	0.0464 (18)	0.070 (3)	0.062 (2)	−0.0275 (19)	0.0155 (17)	−0.032 (2)
N8	0.038 (2)	0.067 (3)	0.052 (2)	−0.014 (2)	0.0108 (18)	−0.028 (2)
C23	0.055 (3)	0.072 (4)	0.070 (3)	−0.009 (3)	0.013 (3)	−0.034 (3)
C24	0.056 (3)	0.077 (4)	0.053 (3)	−0.018 (3)	0.003 (3)	−0.032 (3)
C25	0.038 (2)	0.079 (4)	0.055 (3)	−0.027 (3)	0.015 (2)	−0.035 (3)
O10	0.230 (12)	0.138 (9)	0.169 (9)	−0.105 (9)	0.009 (9)	−0.091 (8)
N9	0.016 (3)	0.079 (5)	0.137 (7)	−0.012 (3)	0.021 (3)	−0.082 (5)
C26	0.025 (3)	0.086 (7)	0.202 (13)	−0.011 (4)	0.008 (5)	−0.102 (9)
C27	0.082 (6)	0.054 (6)	0.077 (6)	−0.025 (5)	−0.019 (5)	−0.006 (5)
C28	0.087 (6)	0.078 (7)	0.079 (6)	−0.055 (6)	0.027 (5)	−0.046 (6)

### Geometric parameters (Å, °)

**Table:**

Cu—N3	1.979 (3)	C13—C14	1.392 (8)
Cu—N3^i^	1.979 (3)	C13—H13	0.9300
Cu—N1	1.992 (4)	C14—C15	1.375 (6)
Cu—N1^i^	1.992 (4)	C14—H14	0.9300
Cu—O1^i^	2.583 (3)	C15—C16	1.395 (7)
Cu—O1	2.583 (3)	C15—H15	0.9300
O1—C9	1.427 (5)	C17—C22	1.373 (7)
O1—C8	1.429 (5)	C17—C18	1.382 (7)
O2—N5	1.227 (6)	C18—C19	1.363 (6)
O3—N5	1.227 (6)	C18—H18	0.9300
O4—N6	1.230 (5)	C19—C20	1.440 (7)
O5—N6	1.228 (5)	C20—C21	1.465 (7)
O6—C20	1.244 (5)	C21—C22	1.366 (6)
O7—N7	1.185 (5)	C22—H22	0.9300
O8—N7	1.199 (6)	O9—C25	1.248 (6)
N1—C7	1.324 (5)	N8—C25	1.306 (6)
N1—C1	1.387 (6)	N8—C23	1.461 (6)
N2—C7	1.342 (6)	N8—C24	1.461 (5)
N2—C6	1.386 (6)	C23—H23A	0.9600
N2—H2N	0.87 (5)	C23—H23B	0.9600
N3—C10	1.329 (6)	C23—H23C	0.9600
N3—C16	1.413 (5)	C24—H24A	0.9600
N4—C10	1.339 (6)	C24—H24B	0.9600
N4—C11	1.389 (6)	C24—H24C	0.9600
N4—H4N	0.87 (5)	C25—H25	0.9300
N5—C17	1.451 (6)	O10—C28	1.266 (7)
N6—C19	1.451 (6)	N9—C28	1.309 (7)
N7—C21	1.456 (7)	N9—C26	1.463 (6)
C1—C2	1.395 (6)	N9—C27	1.468 (6)
C1—C6	1.399 (6)	C26—H26A	0.9600
C2—C3	1.373 (8)	C26—H26B	0.9600
C2—H2	0.9300	C26—H26C	0.9600
C3—C4	1.397 (7)	C27—H27A	0.9600
C3—H3	0.9300	C27—H27B	0.9600
C4—C5	1.374 (7)	C27—H27C	0.9600
C4—H4	0.9300	C28—H28A	0.9300
C5—C6	1.372 (7)	O10A—C28A	1.255 (8)
C5—H5	0.9300	N9A—C28A	1.309 (7)
C7—C8	1.490 (7)	N9A—C27A	1.456 (7)
C8—H8A	0.9700	N9A—C26A	1.466 (7)
C8—H8B	0.9700	C26A—H26D	0.9600
C9—C10	1.489 (6)	C26A—H26E	0.9600
C9—H9A	0.9700	C26A—H26F	0.9600
C9—H9B	0.9700	C27A—H27D	0.9600
C11—C16	1.380 (7)	C27A—H27E	0.9600
C11—C12	1.394 (6)	C27A—H27F	0.9600
C12—C13	1.381 (8)	C28A—H28B	0.9300
C12—H12	0.9300		
			
N3—Cu—N3^i^	180	C14—C13—H13	118.8
N3—Cu—N1	87.55 (15)	C15—C14—C13	121.4 (5)
N3^i^—Cu—N1	92.45 (15)	C15—C14—H14	119.3
N3—Cu—N1^i^	92.45 (15)	C13—C14—H14	119.3
N3^i^—Cu—N1^i^	87.55 (15)	C14—C15—C16	117.1 (5)
N1—Cu—N1^i^	180	C14—C15—H15	121.5
N3—Cu—O1^i^	106.54 (12)	C16—C15—H15	121.5
N3^i^—Cu—O1^i^	73.46 (12)	C11—C16—C15	120.9 (4)
N1—Cu—O1^i^	106.14 (13)	C11—C16—N3	109.3 (4)
N1^i^—Cu—O1^i^	73.86 (13)	C15—C16—N3	129.7 (4)
N3—Cu—O1	73.46 (12)	C22—C17—C18	121.3 (4)
N3^i^—Cu—O1	106.54 (12)	C22—C17—N5	119.6 (5)
N1—Cu—O1	73.86 (13)	C18—C17—N5	119.1 (5)
N1^i^—Cu—O1	106.14 (13)	C19—C18—C17	119.2 (5)
O1^i^—Cu—O1	180	C19—C18—H18	120.4
C9—O1—C8	114.0 (3)	C17—C18—H18	120.4
C9—O1—Cu	105.0 (2)	C18—C19—C20	124.5 (5)
C8—O1—Cu	104.8 (3)	C18—C19—N6	115.8 (5)
C7—N1—C1	105.4 (4)	C20—C19—N6	119.7 (4)
C7—N1—Cu	122.5 (3)	O6—C20—C19	124.9 (4)
C1—N1—Cu	131.4 (3)	O6—C20—C21	123.4 (5)
C7—N2—C6	108.1 (4)	C19—C20—C21	111.7 (4)
C7—N2—H2N	124 (4)	C22—C21—N7	117.0 (5)
C6—N2—H2N	128 (4)	C22—C21—C20	123.6 (5)
C10—N3—C16	104.3 (4)	N7—C21—C20	119.4 (4)
C10—N3—Cu	123.2 (3)	C21—C22—C17	119.5 (5)
C16—N3—Cu	132.4 (3)	C21—C22—H22	120.3
C10—N4—C11	107.3 (4)	C17—C22—H22	120.3
C10—N4—H4N	120 (4)	C25—N8—C23	120.4 (4)
C11—N4—H4N	133 (4)	C25—N8—C24	122.9 (4)
O2—N5—O3	123.3 (4)	C23—N8—C24	116.7 (4)
O2—N5—C17	118.3 (5)	N8—C23—H23A	109.5
O3—N5—C17	118.4 (5)	N8—C23—H23B	109.5
O5—N6—O4	122.7 (4)	H23A—C23—H23B	109.5
O5—N6—C19	119.1 (4)	N8—C23—H23C	109.5
O4—N6—C19	118.2 (4)	H23A—C23—H23C	109.5
O7—N7—O8	120.8 (6)	H23B—C23—H23C	109.5
O7—N7—C21	120.5 (5)	N8—C24—H24A	109.5
O8—N7—C21	118.7 (4)	N8—C24—H24B	109.5
N1—C1—C2	131.0 (4)	H24A—C24—H24B	109.5
N1—C1—C6	109.6 (4)	N8—C24—H24C	109.5
C2—C1—C6	119.4 (5)	H24A—C24—H24C	109.5
C3—C2—C1	118.0 (4)	H24B—C24—H24C	109.5
C3—C2—H2	121.0	O9—C25—N8	124.0 (4)
C1—C2—H2	121.0	O9—C25—H25	118.0
C2—C3—C4	121.0 (5)	N8—C25—H25	118.0
C2—C3—H3	119.5	C28—N9—C26	119.1 (6)
C4—C3—H3	119.5	C28—N9—C27	123.2 (5)
C5—C4—C3	122.1 (5)	C26—N9—C27	117.7 (6)
C5—C4—H4	118.9	N9—C26—H26A	109.5
C3—C4—H4	118.9	N9—C26—H26B	109.5
C6—C5—C4	116.4 (4)	H26A—C26—H26B	109.5
C6—C5—H5	121.8	N9—C26—H26C	109.5
C4—C5—H5	121.8	H26A—C26—H26C	109.5
C5—C6—N2	132.3 (4)	H26B—C26—H26C	109.5
C5—C6—C1	123.1 (5)	N9—C27—H27A	109.5
N2—C6—C1	104.6 (4)	N9—C27—H27B	109.5
N1—C7—N2	112.3 (5)	H27A—C27—H27B	109.5
N1—C7—C8	123.6 (4)	N9—C27—H27C	109.5
N2—C7—C8	124.1 (4)	H27A—C27—H27C	109.5
O1—C8—C7	111.3 (3)	H27B—C27—H27C	109.5
O1—C8—H8A	109.4	O10—C28—N9	116.0 (7)
C7—C8—H8A	109.4	O10—C28—H28A	122.0
O1—C8—H8B	109.4	N9—C28—H28A	122.0
C7—C8—H8B	109.4	C28A—N9A—C27A	126.2 (9)
H8A—C8—H8B	108.0	C28A—N9A—C26A	118.7 (8)
O1—C9—C10	111.0 (4)	C27A—N9A—C26A	115.1 (8)
O1—C9—H9A	109.4	N9A—C26A—H26D	109.5
C10—C9—H9A	109.4	N9A—C26A—H26E	109.5
O1—C9—H9B	109.4	H26D—C26A—H26E	109.5
C10—C9—H9B	109.4	N9A—C26A—H26F	109.5
H9A—C9—H9B	108.0	H26D—C26A—H26F	109.5
N3—C10—N4	113.2 (4)	H26E—C26A—H26F	109.5
N3—C10—C9	123.3 (4)	N9A—C27A—H27D	109.5
N4—C10—C9	123.5 (4)	N9A—C27A—H27E	109.5
C16—C11—N4	106.0 (4)	H27D—C27A—H27E	109.5
C16—C11—C12	122.6 (5)	N9A—C27A—H27F	109.5
N4—C11—C12	131.4 (5)	H27D—C27A—H27F	109.5
C13—C12—C11	115.7 (5)	H27E—C27A—H27F	109.5
C13—C12—H12	122.2	O10A—C28A—N9A	120.0 (10)
C11—C12—H12	122.2	O10A—C28A—H28B	120.0
C12—C13—C14	122.3 (5)	N9A—C28A—H28B	120.0
C12—C13—H13	118.8		
			
N3—Cu—O1—C9	15.0 (3)	C16—N3—C10—N4	0.2 (6)
N3^i^—Cu—O1—C9	−165.0 (3)	Cu—N3—C10—N4	177.0 (3)
N1—Cu—O1—C9	107.2 (3)	C16—N3—C10—C9	178.4 (4)
N1^i^—Cu—O1—C9	−72.8 (3)	Cu—N3—C10—C9	−4.8 (7)
O1^i^—Cu—O1—C9	−79 (100)	C11—N4—C10—N3	0.0 (6)
N3—Cu—O1—C8	−105.5 (3)	C11—N4—C10—C9	−178.1 (5)
N3^i^—Cu—O1—C8	74.5 (3)	O1—C9—C10—N3	19.8 (7)
N1—Cu—O1—C8	−13.2 (2)	O1—C9—C10—N4	−162.2 (4)
N1^i^—Cu—O1—C8	166.8 (2)	C10—N4—C11—C16	−0.3 (5)
O1^i^—Cu—O1—C8	161 (100)	C10—N4—C11—C12	−177.8 (6)
N3—Cu—N1—C7	76.3 (3)	C16—C11—C12—C13	−2.1 (8)
N3^i^—Cu—N1—C7	−103.7 (3)	N4—C11—C12—C13	175.1 (5)
N1^i^—Cu—N1—C7	−42 (100)	C11—C12—C13—C14	0.9 (9)
O1^i^—Cu—N1—C7	−177.2 (3)	C12—C13—C14—C15	0.1 (10)
O1—Cu—N1—C7	2.8 (3)	C13—C14—C15—C16	0.0 (9)
N3—Cu—N1—C1	−92.3 (4)	N4—C11—C16—C15	−175.4 (4)
N3^i^—Cu—N1—C1	87.7 (4)	C12—C11—C16—C15	2.4 (8)
N1^i^—Cu—N1—C1	149 (100)	N4—C11—C16—N3	0.4 (6)
O1^i^—Cu—N1—C1	14.2 (4)	C12—C11—C16—N3	178.2 (5)
O1—Cu—N1—C1	−165.8 (4)	C14—C15—C16—C11	−1.2 (8)
N3^i^—Cu—N3—C10	153 (100)	C14—C15—C16—N3	−176.1 (5)
N1—Cu—N3—C10	−79.7 (4)	C10—N3—C16—C11	−0.4 (5)
N1^i^—Cu—N3—C10	100.3 (4)	Cu—N3—C16—C11	−176.8 (3)
O1^i^—Cu—N3—C10	174.2 (4)	C10—N3—C16—C15	175.0 (5)
O1—Cu—N3—C10	−5.8 (4)	Cu—N3—C16—C15	−1.4 (8)
N3^i^—Cu—N3—C16	−31 (100)	O2—N5—C17—C22	2.5 (7)
N1—Cu—N3—C16	96.1 (4)	O3—N5—C17—C22	−176.7 (4)
N1^i^—Cu—N3—C16	−83.9 (4)	O2—N5—C17—C18	−175.6 (4)
O1^i^—Cu—N3—C16	−10.0 (4)	O3—N5—C17—C18	5.3 (6)
O1—Cu—N3—C16	170.0 (4)	C22—C17—C18—C19	3.9 (7)
C7—N1—C1—C2	−177.3 (4)	N5—C17—C18—C19	−178.1 (4)
Cu—N1—C1—C2	−7.2 (7)	C17—C18—C19—C20	−0.5 (7)
C7—N1—C1—C6	1.0 (5)	C17—C18—C19—N6	−178.4 (4)
Cu—N1—C1—C6	171.1 (3)	O5—N6—C19—C18	−152.7 (5)
N1—C1—C2—C3	179.6 (4)	O4—N6—C19—C18	27.1 (7)
C6—C1—C2—C3	1.5 (6)	O5—N6—C19—C20	29.3 (7)
C1—C2—C3—C4	−1.5 (7)	O4—N6—C19—C20	−150.9 (4)
C2—C3—C4—C5	0.6 (7)	C18—C19—C20—O6	178.7 (4)
C3—C4—C5—C6	0.3 (7)	N6—C19—C20—O6	−3.5 (7)
C4—C5—C6—N2	−178.4 (4)	C18—C19—C20—C21	−2.9 (6)
C4—C5—C6—C1	−0.4 (7)	N6—C19—C20—C21	174.9 (4)
C7—N2—C6—C5	178.2 (5)	O7—N7—C21—C22	167.0 (6)
C7—N2—C6—C1	−0.2 (4)	O8—N7—C21—C22	−10.0 (7)
N1—C1—C6—C5	−179.0 (4)	O7—N7—C21—C20	−13.8 (8)
C2—C1—C6—C5	−0.6 (7)	O8—N7—C21—C20	169.2 (4)
N1—C1—C6—N2	−0.5 (5)	O6—C20—C21—C22	−178.2 (4)
C2—C1—C6—N2	178.0 (4)	C19—C20—C21—C22	3.3 (6)
C1—N1—C7—N2	−1.1 (5)	O6—C20—C21—N7	2.6 (6)
Cu—N1—C7—N2	−172.3 (3)	C19—C20—C21—N7	−175.8 (4)
C1—N1—C7—C8	−179.9 (4)	N7—C21—C22—C17	178.8 (4)
Cu—N1—C7—C8	8.9 (6)	C20—C21—C22—C17	−0.3 (7)
C6—N2—C7—N1	0.8 (5)	C18—C17—C22—C21	−3.5 (7)
C6—N2—C7—C8	179.6 (4)	N5—C17—C22—C21	178.5 (4)
C9—O1—C8—C7	−94.6 (4)	C23—N8—C25—O9	178.3 (5)
Cu—O1—C8—C7	19.7 (4)	C24—N8—C25—O9	−0.3 (7)
N1—C7—C8—O1	−21.8 (6)	C26—N9—C28—O10	0.4 (13)
N2—C7—C8—O1	159.6 (4)	C27—N9—C28—O10	−179.7 (9)
C8—O1—C9—C10	94.0 (4)	C27A—N9A—C28A—O10A	178 (3)
Cu—O1—C9—C10	−20.2 (4)	C26A—N9A—C28A—O10A	−1(5)

Symmetry codes: (i) −*x*, −*y*+1, −*z*+1.

### Hydrogen-bond geometry (Å, °)

**Table:**

*D*—H···*A*	*D*—H	H···*A*	*D*···*A*	*D*—H···*A*
N2—H2N···O6^ii^	0.87 (5)	1.95 (4)	2.709 (5)	145 (6)
N2—H2N···O5^ii^	0.87 (5)	2.41 (5)	3.104 (6)	138 (6)
N4—H4N···O9^iii^	0.87 (5)	1.92 (2)	2.753 (6)	162 (5)

Symmetry codes: (ii) −*x*+1, −*y*, −*z*+2; (iii) −*x*+1, −*y*+1, −*z*+1.
